# Physical and Ski Technical Factors Associated with ACL Injury Susceptibility in Elite and Recreational Alpine Skiers

**DOI:** 10.3390/jfmk11010076

**Published:** 2026-02-13

**Authors:** Márton Kékesi, Dorina Annar, Mira Ambrus, Ádám Uhlár, András Tállay, Zsombor Lacza

**Affiliations:** 1 Research Center for Sports Physiology, Hungarian University of Sports Science, 1123 Budapest, Hungary; kekesi.marton@tf.hu (M.K.);; 2 Department of Sports Medicine, Semmelweis University, 1122 Budapest, Hungary

**Keywords:** alpine skiing, ACL risk factors, dynamic knee valgus, CARV, ski technic, CoRehab

## Abstract

**Introduction**: Anterior cruciate ligament (ACL) injuries are among the most severe and frequent injuries in alpine skiing, often occurring in non-contact situations during high-demand turns. Various instrumental techniques were used to assess susceptibility to anterior cruciate ligament (ACL) injuries in alpine ski racers and recreational skiers. This cross-sectional exploratory study aimed to identify key factors contributing to ACL injury susceptibility, comparing lab-based and on-snow tests. **Materials and Methods**: We examined nine elite ski racers and nine recreational skiers with strong athletic backgrounds. Skiing technique was analyzed using an instrumented insole system (CARV) to measure body position, pressure symmetry, and edge angle. Dynamic Q-angle symmetry during single-leg squats were assessed with an optical system (DynaKnee), while balance, strength, and agility were evaluated through ACL-specific lab tests (CoRehab). Group comparisons were performed using the nonparametric Mann–Whitney U test. **Results**: No significant differences were found between groups in ACL-specific lab tests, including balance, agility, and jump performance. However, ski racers exhibited 34.9% higher asymmetry in the Q-angle symmetry index during the one-leg squat. In contrast, ski technique differences were significant: ski racers achieved 16.3% higher Edge Similarity, 48% better Pressure Symmetry, and 5.8% better Fore-Aft Balance compared to recreational skiers. **Conclusions**: Despite similar general athletic abilities, elite skiers showed higher Q-angle asymmetry, which has been previously associated with ACL injury risk. However, their advanced skiing technique may partially mitigate the functional consequences of this asymmetry during on-snow tests. This suggests that refined skiing skills may influence functional performance in racing conditions, while pronounced one-sided dominance could indicate potential injury risk.

## 1. Introduction

More than one third of alpine skiing injuries are inflicted on the knee and about 40% of them imply the partial or full rupture of the anterior cruciate ligament (ACL) [1]. With the refined technique of ski racers, injuries usually occur in demanding racing or training situations, where correction maneuvers are focused on saving the run even if it implies risking a knee injury [2]. It has been shown that 379 racers in the French Alpine Team suffered an ACL injury during a 25-year period until 2005, and 105 of them required reconstructing surgery at least once. In 40% of the cases, a repeated ACL surgery was performed, and in 20% of these cases, the same knee was injured again [3]. In young and highly active athletes, return to high-demand sports after ACL reconstruction is associated with a markedly elevated risk of reinjury, with approximately 20–25% of athletes sustaining a second ACL injury within the first years after return to sport. Importantly, objective deficits frequently persist at the time of clearance: previous studies have reported that up to 80–90% of young athletes fail to meet combined strength and functional criteria, particularly due to quadriceps strength asymmetries, despite being cleared to return to sport. Moreover, early return to knee-strenuous activities has been shown to substantially increase reinjury risk, with athletes returning before 9 months post-surgery demonstrating up to a sevenfold higher incidence of second ACL injury compared with those delaying return. Therefore, strict objective testing is mandated prior to return to sport, with current evidence recommending achievement of a Limb Symmetry Index (LSI) exceeding 90% in strength and functional performance measures to reduce the risk of reinjury in young active populations [4,5,6,7]. Most of the ACL injuries in skiing happen in non-contact situations, typically during aggressive turning [8].

Understanding the mechanism of the ACL injuries deeper may help preventing them. However, prevention should also focus on the personal susceptibility to this serious injury. This approach may be based on the evaluation of skiing skills and also on specific results obtained by measuring physical characteristics and performance levels. Beyond instrumental detection, the applied measurements also allow the visual observation of individual movement patterns that may be relevant when interpreting potential injury mechanisms [8].

Difficult situations may arise in a racing run if the ski technique performed by the racer is characteristically asymmetric or the key elements of turning are not consistently efficient. Technical shortcomings may, however, be related to physical discrepancies and shortcomings in the major performance types that are required for alpine skiing. Dynamic lower-limb asymmetries, especially valgus collapse, increase ACL strain in pivoting sports including alpine skiing. Laboratory methods quantify these factors, while newer systems allow numerical assessment even during skiing. In this study, we used three instrumental techniques to detect ACL predisposing factors in alpine skiers and to determine which tool best reflects ACL-relevant risk characteristics [9,10,11].

More than 80% of the stress arising from limiting the anterior tibial translating movement is taken by the ACL [12]. This linear stress in the parasagittal plane of the knee and the related hyperflexion had been considered traditionally as the primary cause of ACL ruptures [13]. According to general analysis of motions [14], rotation and valgus shift in the moderately flexed knee may also be important in causing ACL ruptures.

Hyperflexion position of the body resulting in a hyperflexed knee with a wide frontal opening of the knee joint. This is induced by a “backward landing” from a high jump, when the reaction force from the snow acting through the tail of the ski and the back of the boot pushes the tibia forward, while the inertia force acting in the center of mass generates a backward momentum [15,16]. If the rectus abdominis and the quadriceps muscles are not capable of counteracting it, the center of mass falls below knee-height and the knee is hyperflexed, severely stretching the ACL. A similar situation often arises with recreational skiers, who may instinctively drop their hips behind if frightened and let their legs wobble without any control.

However, besides hyperflexion, other extreme positions of the knee by hyperextension, hyper-rotation or a valgus collapse has also become frequent in ski racing to cause ACL ruptures. Trauma is generally related to the forceful opening of the gap between the condyle of the femur and the tibia. It may happen symmetrically or—if combined with torsion—asymmetrically in the anterior or the posterior sides.

Knee hyperextension arises when the skier is pushed forward with straight legs by an uncontrolled momentum. The hyper-valgus position of the knee accompanied by a hyper-rotation is more likely to happen to racers on icy racing tracks, when the outside ski slips and the body weight is dropped on the inside ski which is often caught on the edge, thereby straining the ACL. These kinds of situations are technically termed as “slip and catch”, when the body hangs behind the natural position and the outside edge of the inside ski is caught by the snow, or “dynamic snowplow”, if this arrangement is in the opposite way. Although the stresses in the knee caused by a valgus shift is borne by the medial collateral ligament [17,18], the valgus and rotational load of the knee may significantly increase the danger of ACL injury [19,20].

Females usually bear loads with more pronounced valgus angles, which results in higher vulnerability to knee injuries. However, in general, deficits in the strengths of the quadriceps, biceps femoris and the gluteus muscles may also lead to injuries of the knee ligaments. This dynamic valgus tendency of the knee is often measured in movement analysis labs during single-leg squats or single-leg landings [9,21], allowing a screening method for skiers to evaluate ACL injury risk before it happens. Previous research has demonstrated that individuals with higher static Q-angles tend to exhibit greater dynamic knee valgus during single-leg squat tasks, indicating a link between frontal-plane alignment and functional knee control [22]. Furthermore, altered activation timing between the vastus lateralis and vastus medialis has been associated with increased dynamic valgus in female athletes [23].

Laboratory-based screening of ACL-relevant factors typically evaluates frontal-plane knee alignment, lower-limb symmetry, and neuromuscular performance [21]. Dynamic Q-angle during functional tasks is used to characterize valgus tendency under load [23], and neuromuscular factors have been linked to dynamic knee valgus in single-leg tasks [22], while systems such as CoReHab assess unilateral balance and coordination in a controlled environment. Such assessments are also used in return-to-sport decision-making, where balance, agility, strength and coordination tests are commonly employed, often expressed through symmetry indices, as asymmetry is considered an important indicator of injury risk [24]. The CARV insole is a smart electronic device which may provide objective measurements for the basic technical features of skiing. The expert eye can see the mistakes in skiing that may result in knee injuries if it hinders efficient execution of the technique in difficult racing situations.

However, these tests are performed in a controlled laboratory setting, and it remains unclear how they relate to skiing-specific movements. Recent model-based simulations have provided insights into ACL loading during alpine skiing turns, highlighting the unique mechanical demands acting on the knee during aggressive maneuvers [10]. As noted by Spörri et al. [11], real-world biomechanical assessment in snow sports remains challenging, and laboratory metrics do not fully replicate on-slope loading conditions. Although peer-reviewed validation of some field-based smart insole technologies is still limited, their use allows the assessment of skiing-specific performance directly on snow under ecological conditions that cannot be reproduced in laboratory settings.

The aim of the current study was to compare ACL-relevant laboratory- and on-snow-derived movement parameters between licensed alpine ski racers competing in Fédération Internationale de Ski (FIS) and recreational skiers, and to explore whether group differences observed in laboratory-based assessments are reflected in skiing-specific performance measures. We hypothesized that FIS alpine ski racers and recreational skiers would differ in both laboratory-based symmetry measures and skiing-specific technical parameters, and that on-snow assessments would reveal technique-related asymmetries not detectable in laboratory tests.

## 2. Materials and Methods

### 2.1. Participants

The examined subjects were divided into two groups, age between 18 and 30 years, the first consisting of 9 FIS racers in the National Team of Hungary with advanced international racing experience, while the 9 remaining subjects represented recreational skiers with regular skiing practice, high general physical fitness, and no competitive racing background or professional alpine ski training. The sample included both male and female participants. All participants reported a minimum of 10–15 years of skiing experience, with elite racers training, on average, 80 days per year and recreational skiers 10 days per year. Exclusion criteria included lower-limb injury within the past 12 months. Two participants had undergone ACL reconstruction more than two years prior to testing and were fully rehabilitated, asymptomatic, and actively training/racing at the time of data collection. Elite ski racers were recruited from the Hungarian national alpine skiing program, while recreational skiers were recruited as a convenience sample based on availability and willingness to participate. Although this study includes a limited sample of skiers, this is primarily due to the small national pool of high-performance alpine skiers in Hungary from which eligible participants could be recruited, resulting in a necessarily narrow selection base. Furthermore, the study design required a complex measurement protocol involving both laboratory-based assessments and on-snow testing under controlled environmental conditions, which further limited feasible sample size. The local ethics committee approved the study (MTSE-KEB/No21/2024), which was conducted in accordance with the most recent version of the Declaration of Helsinki, and all participants provided written informed consent prior to participation.

### 2.2. Complex Measuring of Dynamic Performance in the Lab

**DynaKnee**

**Figure 1:**
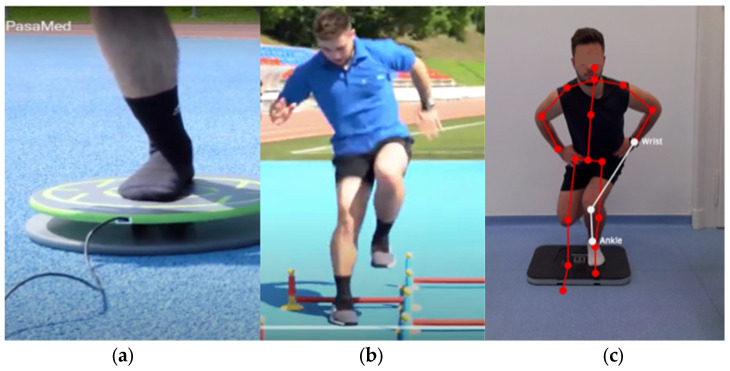
Laboratory tests modeling ski turns. Panel (**a**) shows the one leg stability in the CoRehab protocol. Panel (**b**) shows the Parkour test with one leg in the CoRehab protocol. Panel (**c**) shows knee Q-angle, valgus and varus ratio during the single-leg squat. Source: Author’s own.

Measuring virtual Q-angles as an indirect indicator of dynamic knee alignment, the change in the quadriceps (Q) angle of the knee—describes the frontal-plane angle formed between the quadriceps muscle force vector and the patellar tendon, reflecting medial–lateral loading alignment at the knee joint—between the relaxed stance and single-leg squat was detected by the DynaKnee system (Orthosera Medical, Budapest, Hungary) as shown in Figure 1. Increasing loads on the bent leg triggers a commonly associated motion of letting the knee shift into deeper valgus positions. This method has been previously shown to exhibit acceptable reliability for assessing frontal-plane knee alignment during single-leg tasks [9,25]. Dynamic increases in Q-angle during functional tasks are thought to reflect the combined effects of frontal-plane alignment and neuromuscular control demands, particularly under single-leg loading [22,23]. The system was extensively evaluated and has been previously validated for dynamic knee valgus assessment in earlier studies, and the most accurate and reproducible variable describing dynamic Q-angle behavior was the medial shift in the knee joint, expressed as a percentage of the lower-limb length, which was therefore used for further analysis [9]. During the single-leg squat task, three squats were performed on each limb, and the value corresponding to the maximum squat depth was used for analysis. This deepest point was defined relative to the single-leg stance starting position. Short, self-selected rest (~30–60 s) was allowed between limbs.

**CoRehab—Back in Action**

Skiing and ACL-specific performance characteristics, e.g., balance coordination, dynamic strength, power endurance, quick power, and static strength were evaluated by the “Back in Action” (BIA) program from CoRehab (CoRehab s.r.l., Trento, Italy). Components of the CoRehab system ensure the accurate detection of limb asymmetries and fatigue-related changes, as other articles have shown [26,27]. The following tests and indicators were used to assess the abilities of skiers with relevance to the susceptibility of a knee injury:

- **Stabilometric balance:** quantified as total off-balance time (s), representing the cumulative duration during which the center of pressure exceeded predefined stabilometric thresholds.
- **Vertical jump (CMJ)-single-leg and double-leg:** height and jump power normalized to body mass (W/kg). CMJ height was calculated using the flight-time method. Flight time was derived from the vertical acceleration signal recorded by the IMU sensor, and jump height was computed using the Bosco equation.
- **“Parkour” obstacle course:** time of completion (s) in multidirectional jumps with single-leg.
- **“Quick feet”:** completion time (s) of rapid alternating foot contacts on predefined targets.

The execution of these tests is shown in Figure 1. The CoRehab test battery was executed in the following order: (1) Stabilometric balance, (2) CMJ, (3) Parkour, (4) Quick Feet.

Short self-selected rest periods (~30–60 s) were allowed between tests to avoid fatigue accumulation.

Test execution and repetitions were standardized as follows:

- **Stabilometric balance:** 2 trials of 15 s each, mean value used for analysis;
- **CMJ single-leg and double-leg:** 3 trials per condition, mean value calculated **per leg** in the single-leg condition;
- **Parkour:** 2 trials **per leg**, mean value **per leg** used for analysis;
- **Quick Feet:** 2 trials **per leg**, mean value **per leg** used for analysis.

In addition to absolute performance values, symmetry indices were calculated for each measure as defined in Equation (1).

### 2.3. Measurements on the Ski Slope

**Figure 2:**
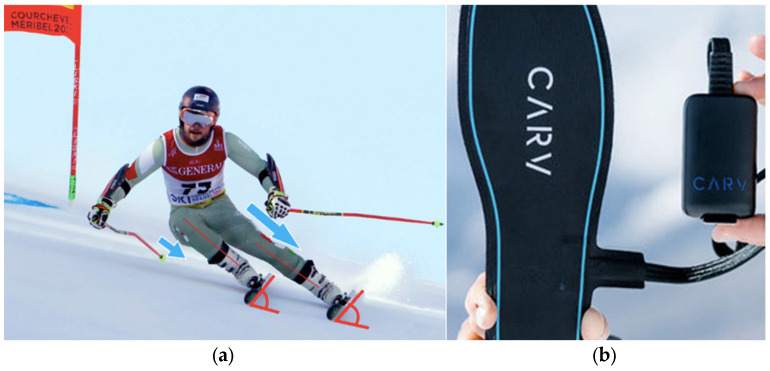
On-snow test system. Panel (**a**) shows illustrates measured foot pressure force (blue arrows) and edge angle (red lines) and fore/aft pressure (black lines) during a race turn. Panel (**b**) shows the CARV system, a skiing-adapted pressure mat and accelerometer that collects and analyses skiing data on piste. CARV footplate is inserted under the inner boot. Source: Author’s own.

The CARV smart insole system has been used in elite skiing environments and is increasingly applied for technique assessment in the field. However, as a relatively new technology, published peer-reviewed validation data are still limited. In the current study, we used the CARV digital ski coach system (Carv by Motion Metrics Ltd., UK) consisting of a sensor-laden insole fitted below the ski boot liner that sends pressure distribution and accelerometric data to the main unit fixed at the outside cuff of the ski boot. The two units are linked with a direct data cable, as shown in Figure 2b, and data are continuously transferred through Bluetooth connection to a mobile phone carried by the skier and running a dedicated data capture and evaluation application. The skiing technique performed is expressed from data recorded in the turns and converted into 12 numerical characteristics describing the amount, the change and the distribution of pressure on the skis, the directional and angular positions of the skis, as well as the relevant similarity and symmetry parameters. In the current study, we selected three variables from the CARV recordings that are relevant for ACL strain:

- fore/aft pressure, indicating position on the ski especially backward drift;
- left/right pressure, mainly to monitor movement asymmetry;
- edge angle symmetry, the difference of which reflects dynamic valgus during ski turns.

CARV-derived variables represent proprietary composite indicators based on pressure distribution and inertial sensor data and were interpreted as technique-related descriptors rather than direct biomechanical measures.

The execution of these tests is shown in Figure 2, and the selected ACL-specific measures are summarized in Table 1.

Snow conditions, measuring methods: It was performed on a groomed piste, red-category slope under stable winter conditions (−2 to 0 °C, no precipitation, minimal wind) in early March. Participants completed 2–3 ski runs, and a representative run was selected for analysis based on stable and technically correct execution, as evaluated by an experienced alpine ski coach.

The direct values obtained by the three different systems give some basic characteristics for the physical abilities of the persons tested. Further, the limb symmetry can be expressed by the ratio of the single or average values (of the same sign) yielding the symmetry index (S.I.):

(1) $$S.I.\;=\;\frac{{\bar{y}}_{LS}}{{\bar{y}}_{HS}}\times 100\%,\;\;\;{\bar{y}}_{LS}<\;{\bar{y}}_{HS}$$

where $\bar{y}$ stands for the (average) measured values, and the side of the lower value is denoted by the LS subscript, whereas HS stands for the side of the higher (average) value. However, some systems (e.g., CoRehab stability) may use only the shortest times from the repeated tests for expressing a modified symmetry index (M.S.I.):

(2) $$M.S.I.\;=\;\frac{{\left(\min_{i}y_{i}\right)}_{LS}}{{\left(\min_{i}y_{i}\right)}_{HS}}\times 100\%$$

The standard indices expressed by Equation (1) or their transformations, based on singular or average values, are used for the evaluations further on.

**Instrument validity and reliability considerations:**

The three measurement systems used in this study differ in their methodological purpose and level of peer-reviewed validation. The DynaKnee system provides non-invasive, markerless estimation of frontal-plane knee alignment and has been validated for assessing dynamic knee valgus during single-leg tasks in athletic populations [9]. The CoRehab exergaming platform has been applied in sports and rehabilitation contexts to assess balance, unilateral coordination, and performance symmetry, with studies reporting acceptable test–retest reliability for balance-oriented tasks. In contrast, CARV is primarily designed as a skiing technique assessment tool rather than a clinical biomechanical system, and peer-reviewed validation of its composite metrics (e.g., edge symmetry, pressure distribution) remains limited; therefore, CARV-derived variables in the present study were interpreted as performance descriptors rather than direct biomechanical measures.

### 2.4. Statistics

To identify differences in the studied parameters between FIS racers and recreational skiers, we conducted a nonparametric Mann–Whitney U test due to the small sample size. Effect sizes were assessed using Cliff’s delta, with the following interpretation: negligible: <0.15; small: 0.15; medium: 0.33; large: 0.47 [28]. The statistical power of the Mann–Whitney U test was estimated using a Monte Carlo simulation approach based on the observed Cliff’s delta effect sizes, following previously described simulation-based methods [29]. Synthetic datasets were generated assuming a fixed sample size of nine participants per group, and the proportion of statistically significant results was calculated over 1000 simulation runs (α = 0.05). Statistical analyses were performed in R (version 4.3.3), with hypotheses tested at the 5% significance level.

## 3. Results

### 3.1. Complex Measurement of Dynamic Performance in the Lab

**Figure 3:**
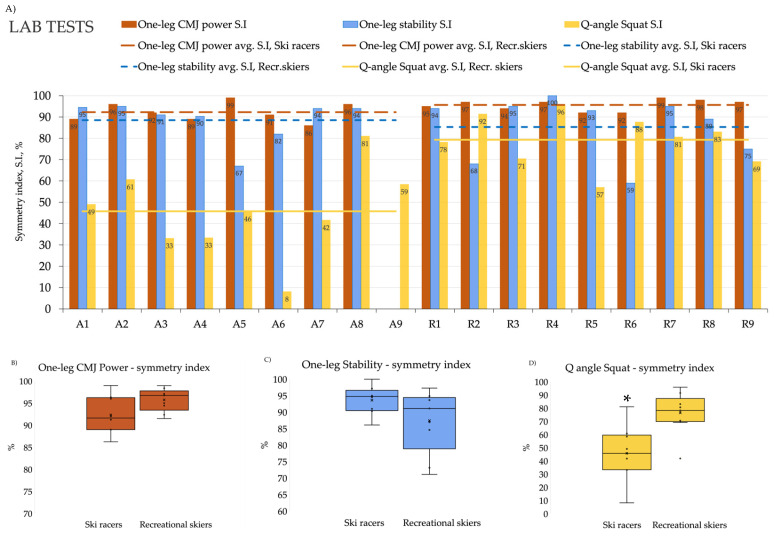
Results of lab tests between A = athletes and R = Recreation skiers in skiing-specific tasks. Panel (**A**) shows the symmetry indices of all subjects; respective averages are shown as trend lines. Note that the recreational skiers scored just as well as the professionals in One leg CMJ Power and Stability tests, and even better in the Dynamic Q-angle Squat test. The One leg CMJ Power and Stability tests (Panels (**B**,**C**)) are slightly better in the recreational skiers. Interestingly, the Q-angle Squat symmetry index reflecting knee alignment under single-leg loading was significantly higher (i.e., more symmetric) in the recreational cohort (panel (**D**)) compared to elite racers. Asterisk indicates *p* < 0.05.

The Q-angles measured in the positions of a single-leg squat were found to be about 10° (~44%) higher than in the relaxed two-legged stance, as an average, indicating that there is a general valgus tendency with flexed knees when the loading is on a single leg, which is a typical feature of skiing (Figure 2). We found significant difference in the Q-angle symmetry index, with elite racers demonstrating 34.9% greater asymmetry compared to recreational skiers (*p* = 0.002, large effect size), indicating increased side-to-side differences in knee alignment at the deepest point of the single-leg squat (Figure 3, Table 2). Overall, ski racers were less symmetric in this variable than well-trained recreational skiers.

With the CoRehab system, we measured basic abilities such as balancing, explosive power, coordination and speed. Our recreational skier’s cohort scored just as well as the professional skiers, confirming that the selected group contained well-trained athletes, and the main difference between the two groups is their skiing skill level and not general athletic performance. In CMJ Power and Stability symmetry tests, recreational skiers scored slightly better than ski racers (approximately 5% and 4%, respectively), although these differences were not statistically significant. Symmetry indices from these lab tests showed a high level for each athlete and both groups on average (Figure 3, Table 2). At the individual level, one ski racer with a recent history of ACL reconstruction who had already returned to competitive skiing still demonstrated interlimb asymmetry values exceeding 10% in CMJ Power Symmetry performance measure.

### 3.2. Measurements on the Ski Slope

**Figure 4:**
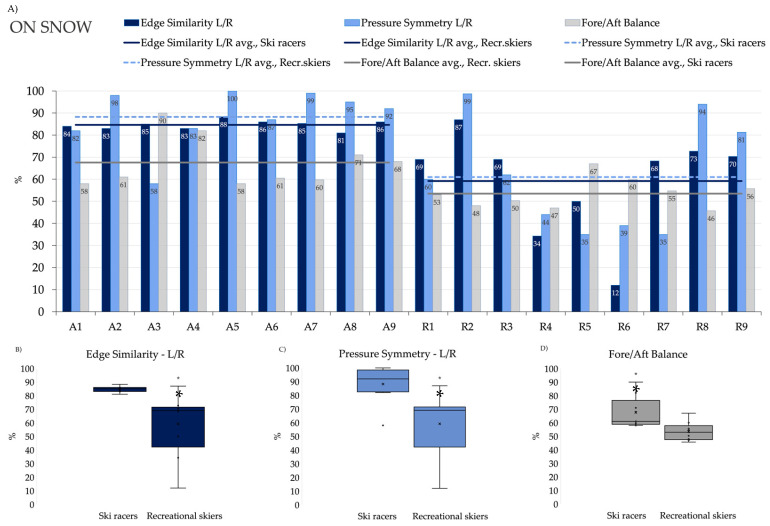
Results of on-snow tests between A = athletes and R = Recreation skiers measured by the CARV boot insert. Panel (**A**) shows the symmetry indices of all subjects; respective averages are shown as trend lines. Note that Edge Similarity and Pressure Symmetry indices were consistently higher in elite racers than in recreational skiers (Panels (**B**,**C**)) and are close to 100% in professionals, while significantly lower in recreational skiers, as expected from their less refined technique. Fore/Aft Balance also shows that professionals are significantly more forward on their skis than recreational skiers (Panel (**D**)). Asterisk indicates *p* < 0.05.

However, on-snow tests measured with the CARV system showed a different picture. The pressure and stability symmetry, that reflects the similarity of left and right turns and performance of left and right legs, within those turns was highly symmetric in racers, while far less so in the recreational group (Figure 4, Table 2). Edge Similarity—which corresponds to Q-angles in a single-leg squat—was significantly higher in elite racers than in recreational skiers, with an absolute median difference of 16.3% (*p* = 0.002, large effect size). Furthermore, ski racers exhibited 48% better Pressure Symmetry and significantly better Fore/Aft Balance by 5.8% (both *p* < 0.01, large effect sizes) compared to recreational skiers, highlighting a clear difference in technical skiing abilities.

## 4. Discussion

The main observation of the current study is that lab tests do not correspond well with on-snow tests in ACL-specific variables; however, both provide valuable insights. From a battery of ACL-specific lab tests, only the dynamic Q-angle test assessment indicated potential asymmetries which, in our racers, were even more pronounced than in well-trained non-skiers. However, these same racers were able to compensate for this issue on snow the least during casual turning, while the generally well-trained but inexperienced skiers showed just the opposite: in contrast to better lab tests, they exhibited marked imbalances and asymmetries during skiing. Since both groups are prone to ACL injuries, these findings should be interpreted as exploratory and descriptive rather than causal, and they suggest that lab-based and on-snow assessments may capture different aspects of ACL-relevant movement patterns.

During a single-leg squat, increasing eccentric load can alter coordination among quadriceps components. The central muscles, vastus intermedius and rectus femoris play a major role, but the vastus lateralis (VL) and vastus medialis obliquus (VMO) also contribute to knee stabilization, with the VMO’s medial orientation influencing dynamic valgus. Therefore, an increasingly dominant VM action may contribute in some degree of valgisation. Valgisation is a result of such muscle mechanics during common everyday movements and sport-specific tasks and may play a role in attenuating axial loads and sudden impacts on the leg by allowing a longer stabilization path. However, excessive or asymmetric valgus alignment may also reflect suboptimal neuromuscular control. This shift in knee position may be more pronounced if the VL and Glutes is less-trained, especially compared to its counterpart muscles [22,25,30]. Thus, the increment in the Q-angle S.I can show the conditions of the quadriceps control of frontal-plane knee alignment in relation to ACL protection, which is of primary importance for ski racers [31]. Interestingly, the racer cohort in our study fared worse than expected in the lab tests for dynamic valgus, highlighting that although skiing does require a strong muscle balance and compensation of valgisation, this level is not achieved through classical training techniques; at least, this was the case in our participants. A significant asymmetry in the Q-angle S.I change for the left and the right legs means different physical and functional abilities of the two legs. This asymmetry may indicate a potential vulnerability for ACL injury under racing circumstances where difficult situations may arise also in the turns where the weaker limb is dominant. Pressure distribution and edge angles between the two skis during turns can be used as the basis for the technical characterization of skiing. The execution of the turns, the positions of the body and the skis, as well as the exerted forces, determine the physical load on the knee and the ability to avoid dangerous situations. The CARV insole system provided valuable insights into the skiing technique of both professional and recreational skiers that are relevant to ACL load and comparable to lab-based tests. Notably, exergaming-based neuromuscular training systems such as CoRehab have been shown to facilitate improvements in motor coordination, balance control, and task engagement in both athletic and rehabilitation contexts, which may partially explain the relatively symmetric neuromuscular performance observed in both groups during laboratory testing.

The “Fore-Aft balance” of the body along the longitudinal axis of the skis strongly affects the tensile stress in the ACL. The ideal stance is at 55–60%, slightly forward relative to the normal (50%) position. Shifting the body far back would not just stress the ACL but may also completely terminate the ability of the skis to carve the arc of the turn. On the other hand, a sudden forward shift in the body may result in a jerky turn, and on a rough terrain, it may also cause the hyperextension of the knee. We found significant differences between ski racers and recreational skiers in the measured Fore-Aft Balances—this means that the ski racers in the examined group were more forward and mostly in the optimum range or slightly forward of it. In the recreational skiers, only one person produced a positive difference, and all the recreational skiers were found falling behind the ideal range tending to induce higher tensile stresses in the ACL and spoiling the turning ability of the skis. This variable cannot be reliably modeled in a laboratory setting, so skiers can and should be tested and trained on snow for the correct Fore-Aft Balance position.

Pressure distribution between the two sides should be symmetrical in the turns of the opposite directions. This is critical for a safe run along the entire racing course. We found significant differences in Pressure Symmetry among the two groups, where the recreational skiers exhibited substantially greater asymmetry than the ski racers. Interestingly, this rather large asymmetry was not at all observable in lab tests, i.e., jumps or squats that somewhat mimic ski turn movements. Similar results were obtained with edge angles of the left and the right skis. Racer athletes’ skis are held at similar edge angles throughout the turns. However, the measurements of the recreational skiers show extremely low symmetry indices, probably caused by their typical habit of keeping the inside ski mostly flat on the snow, using it only as a platform for obtaining vertical support. Given the cross-sectional group-comparison design, the present study does not test compensatory mechanisms directly, nor does it establish cause–effect relationships. The observed group differences should therefore be viewed as skiing-specific performance characteristics rather than biomechanical adaptations. Sports performed in a dynamically valgus knee position on rough terrain by recreational skiers is indeed a high-risk exercise even in those individuals who otherwise exhibit good valgus compensation in more common tasks in the lab. Therefore, this comparison between lab- and on-snow tests shows, again, that field measurements are far more relevant in this regard than any lab- or gym-based tests alone.

This study has several limitations that should be acknowledged. First, its cross-sectional design compared two groups at a single time point and therefore does not allow conclusions regarding injury prediction, ACL loading, or causal relationships between laboratory measures and skiing performance. In addition, the absence of longitudinal follow-up precluded evaluation of return-to-sport timelines and recovery trajectories. Second, the sample size was relatively small due to the limited national pool of elite alpine skiers available for recruitment and the dual laboratory/on-snow testing protocol, which restricts statistical power and generalizability. Third, although all measurement tools used in this study are applied in elite sport settings, comprehensive peer-reviewed validation data for all CARV-derived metrics are still limited. Fourth, on-snow assessments were conducted under controlled free-skiing conditions rather than competition settings, and environmental factors such as snow quality and slope gradient may have influenced performance despite standardization.

Future studies should therefore include larger and more diverse samples, prospective follow-up to evaluate injury incidence, and multi-site validation under competition-like conditions in order to determine whether the observed characteristics are relevant for ACL injury risk in real-world environments.

## 5. Conclusions

This exploratory study highlights the importance of considering both physical and skiing-specific factors when assessing ACL injury susceptibility in skiers. Laboratory assessments indicated greater knee asymmetry in professional racers, while on-snow measurements showed that skilled athletes could compensate for such asymmetries through their technique, whereas recreational skiers exhibited imbalances relevant for ACL loading. These findings highlight that laboratory-based asymmetry screening alone does not fully reflect skiing-specific knee loading, and that combining laboratory and on-snow assessments provides a more realistic evaluation of ACL-related risk factors. Advanced testing methods can aid in improving ski technique and evaluating readiness to return to sport after ACL reconstruction.

## Figures and Tables

**Table 1:** The measured CARV characteristics relevant to the vulnerability for ACL injuries. * Average values of the best ski instructors Source: Author’s own.

Characteristic	Correlation with Stability and Biomechanics	Optimal *
Fore-Aft Balance	The position of the center of mass relative to the center of the ski boot influences the basic tension of the ACL and it can improve or impair the balance and the control of the run.	55~60%
Pressure Symmetry	The symmetry of pressure distribution on the two skis referring to the left and right turns is in correlation with the biomechanical symmetry.	>90%
Edge Similarity	The similarity of the edge angles of the two skis indicates stability and the avoidance of the knee positions endangering the ACL.	>90%

**Table 2:** Descriptive statistics of laboratory- and on-snow-derived variables in elite ski racers and recreational skiers.

Variable	Ski Racers (n = 9) Median	Recreational Skiers (n = 9) Median	*p*-Value	Effect Size (Cliff’s δ)	Power (n = 9 per Group)
Edge similarity (%)	85.3 (83.0–86.0)	69.0 (50.0–70.3)	0.002	0.802	0.940
Pressure symmetry (%)	92.0 (83.0–99.0)	44.0 (35.0–81.3)	0.024	0.629	0.644
Fore–aft balance (%)	60.5 (58.0–71.0)	54.7 (47.0–60.0)	0.002	0.802	0.940
Q-squat S.I (%)	45.8 (33.2–58.5)	80.7 (70.5–91.5)	0.002	0.827	0.953
Stability S.I (%)	95.0 (90.3–97.0)	91.0 (85.0–95.0)	0.158	0.416	0.286
One-leg CMJ Power S.I (%)	92.0 (89.0–96.0)	97.0 (94.0–99.0)	0.078	0.513	0.418

Note: Values are presented as median and interquartile range (IQR). Group comparisons were performed using the Mann–Whitney U test. Effect sizes are reported as Cliff’s delta. Percentage differences reported in the text represent absolute differences between group median values on a 0–100 scale.

## Data Availability

The original contributions presented in this study are included in the article. Data are available upon reasonable request from the corresponding author.

## Abbreviations

The following abbreviations are used in this manuscript:

ACL	Anterior Cruciate Ligament
CMJ	Countermovement Jump
VL	Vastus Lateralis
VM	Vastus Medialis
VMO	Vastus Medialis Oblique
S.I.	Symmetry Index
FIS	Fédération Internationale de Ski
Q-angle	Quadriceps angle
BIA	Back in Action Test
M.S.I.	Movement Symmetry Index
LS	Lower side
HS	Higher side
